# Finite Element Analysis of Flexural Behavior of Shape Memory Alloy Hybrid Composites Laminates

**DOI:** 10.3390/polym14235268

**Published:** 2022-12-02

**Authors:** Lidan Xu, Jitao Zhao, Xiangyu Zhang, Mingfang Shi, Zhenqing Wang

**Affiliations:** 1 School of Civil Engineering, Inner Mongolia University of Science and Technology, Baotou 014010, China; 2 School of Civil and Architecture Engineering, Panzhihua University, Panzhihua 617000, China; 3 College of Aerospace and Civil Engineering, Harbin Engineering University, Harbin 150001, China

**Keywords:** SMAHC, finite element model, flexural properties, optimal content, optimal position

## Abstract

In this paper, experiments and finite element analysis methods were adopted to study the flexural performance of shape memory alloy hybrid composites (SMAHC). The effect of embedding content and position of shape memory alloy (SMA) wire on the flexural properties of composite laminates was analyzed, and the optimal content and position of SMA wire were obtained. The optimal number of SMA wires for SMAHC laminates (B-2) is four. Compared with GFRP laminates, the flexural modulus of B-2-4 laminates increases by 5.19%, while the strength decreases by 5.76% on average. The finite element model of the SMAHC laminate was established by using ABAQUS finite element analysis software, and the validity of the model was verified by the comparison between the simulation results and the experimental results. The microscopic morphology results show that the weak interface between the matrix and SMA has a certain influence on the flexural performance of SMAHC specimens.

## 1. Introduction

Smart materials and structures have brought forth a paradigm shift in design, mostly to the fields of structural engineering, robotics, and aerospace, due to which the demand for highly functional and lightweight, adaptive systems is continuously growing [1,2]. Shape memory alloys (SMAs) are a unique class of metal exhibiting two fundamental properties, namely, superelasticity (SE) and shape-memory effect (SME) [3,4]. Due to the two special characteristics, SMAs have been successfully commercialized in the aerospace, medical, electrical, and oil industries.

The fiber-reinforced resin-based composite material is embedded with SMA; the composite material can acquire the ability to adapt to the environment under the control of the induction unit [5,6]. Due to the excellent superelasticity and shape memory effect, the performance of low-speed impact resistance, damage resistance, vibration, noise reduction and flexural resistance can be significantly improved by embedding shape memory alloy into the composites [7,8]. 

Many scholars have systematically studied the shape memory effect. Lagoudas and Tadjbaksh [9] studied the bending performance of a flexible rod embedded with SMA wire, obtained the deformation shape of a flexible rod, and determined the critical value of phase transition-induced strain. Auricchio et al. [10] used numerical methods to study the shape memory effect of SMA beams under complex stress conditions and calculated the deformation of beams under bending and thermal cycling loads. Lal and Markad [11] considered the finite element governing equation-based higher order shear deformation theory with the principle of virtual work. They used micromechanical methods to calculate the mechanical properties of composite plates, revealing the effects of SMA volume fraction, restoring stress, presence or absence of SMA layers, and temperature on critical buckling loads. Lu et al. [12] presented a theoretical model for the bending of a laminated beam embedded with an SMA fiber-reinforced layer. They discussed the bending of the laminated beam and provided the relationship between bending moment, curvature and temperature. Viet and Zaki [13] proposed a bending model for a laminated composite cantilever beam with multiple embedded SMA layers presenting tensile–compressive asymmetry. They highlighted the necessity of considering asymmetry in the accurate simulation of laminated beams with SMA layers. Tsukamoto [14] investigated the mechanical behavior of NiTi long-fiber reinforced aluminum (Al) matrix composites, experimentally and numerically. It was concluded that internal stresses in the composites, which were introduced during the fabrication processes, highly affected the subsequent mechanical behavior of the composites. Zhang et al. [15] prepared epoxy resin matrix composites filled with NiTi alloy staple fibers and particles and studied their mechanical properties. Due to the addition of SMA filler, the flexural stiffness of SMA/ epoxy composites is increased. Experimental results show that adding only 3.5% SMA filler to epoxy resin results in a significant increase in energy storage modulus, which is six times larger than the epoxy resin body (ER3). Scholars’ research mainly focused on the control of SMA on the bending deformation of its composites and the influence of SMA short wires and particles on the bending properties of composites. The influence of SMA content and position on the overall properties of the composite was not considered.

In terms of superelastic research, Viet and Zaki [16] propose a novel and simple exact semi-analytical model for superelastic SMA wire-reinforced composites subjected to bending loads. The composite is found to behave superelastically under certain conditions of temperature, SMA volume fraction, and elastic stiffness of the matrix. Viet et al. [17] also proposed a new analytical model for a superelastic shape memory alloy prismatic cantilever beam subjected to a concentrated force at the tip. Analytical moment curvature and shear force–shear strain relations are also derived during the loading and unloading of the beam. Pattar et al. [18] researched the mechanical properties of glass/epoxy composite by embedding shape memory alloy and nanoclay. Results show that the flexural strength of SMA/MMT/glass/epoxy composite was improved by 21%, when it was compared with composite with glass/epoxy composite. Aslani et al. [19] used four different codes to calculate the flexural toughness of the shape memory alloy, polypropylene and steel fiber-reinforced self-compacting concrete. It is found that the shape memory and superelasticity of NiTi SMA are effective in delaying initial crack formation and limiting crack width. Pazhanivel et al. [20] adopted the method of embedding nickel–titanium SMA in the cross-section below the neutral axis of glass fiber-reinforced polymer (GFRP) composites to enhance the bending resistance of the composites. Under static loading condition, up to 4 wt% of shape memory alloy reinforcement, the GFRP exhibit enhanced flexure resistance. Katsiropoulos et al. [21] improved the damping behavior of fiber reinforced polymer composites with embedded superelastic SMA. It was found that the addition of Ni-Ti wires into the aramid/epoxy composite leads to a significant modification of its damping, which depends strongly on wire volume fraction. Taheri-Behrooz et al. [22] developed research to predict stress transfer and interfacial behavior between shape memory alloy wire, interphase, and matrix. Inhomogeneous interphase and different radial and hoop stress components in each phase are considered to achieve deeper physical understanding. Khalili et al. [23] investigated the flexural properties of sandwich composite panels with glass laminate aluminum-reinforced epoxy facesheets strengthened by SMA wires. The results represent the advantage effect of shape memory alloy wires on the behavior of sandwich composite panels in bending. Although there have been many studies on the influence of SMA content on the mechanics of composite materials, the current researches on the influence of SMA wire embedding position and content on SMAHC laminates and the finite element modeling of SMAHC laminates are not detailed enough.

Debski [24] and Wysmulski [25] studied the influence of eccentric compression load on the stability and bearing capacity of thin-walled composite profiles by experimental study and finite element method. They investigated the effect of laminate layup and cross-sectional shape on the buckling load, postbuckling equilibrium path and failure load of the structures under compression. Numerical postbuckling equilibrium paths were used to estimate the critical loads with an approximation method. The experimental results were used to validate the numerical models. Then, Rozylo and Wysmulski [26] analyzed the phenomenon of failure of axially compressed thin-walled composite structures with channel shapes. They carried out experimental tests and advanced finite element numerical calculations on actual columns. The cohesive zone model (CZM) was used in numerical studies. The obtained results of the numerical tests showed a high compliance with the results of the experimental studies. Han et al. [27] used the finite element analysis (FEA) method to simulate the mechanical behaviors of ultrastrong and tough bulk material under a tensile state. FEA results showed that water molecules played an active role in fabricating strong and tough materials, by plasticizing and forming hydrogen bonding among cellulose nanofibrils. Hu et al. [28] studied the effect of loading type on mechanical properties, including elastic constants, yield strength and ultimate strength of beech wood. The results show that the finite element models proposed can predict the mechanical behaviors of wood in tensile and compressive states. Valachova and Skotnicova [29] focused on the thermal engineering assessment of light building envelope structures using the finite element method (FEM). The results demonstrate that it is possible to predict the thermal behavior of lightweight peripheral buildings with the help of appropriate simulation programs. The research results of scholars, such as fiber toughening methods, the establishment of models and boundary conditions, and the setting of cohesion contact, have good guidance for this paper.

Recently, research interests in using superelasticity SMAs have extended to civil engineering, where particular effort was devoted to the mitigation of seismic effects on structures [30]. Fang et al. [31] presented a novel type of shape memory alloy (SMA) cable-restrained high damping rubber (SMA-HDR) bearing. The application of the novel SMA-HDR bearing can significantly alleviate the pounding effect, especially under near-fault earthquakes. Chen et al. [32] proposed a novel type of self-centering damper employing NiTi shape memory alloy (SMA) bars. Compared with the existing types of SMA-based dampers, the proposed damper offers a tunable hysteretic behavior with amplified deformability and hybrid sources of energy dissipation. Fang et al. [33] introduced a new type of shape memory alloy (SMA) washer spring-based self-centering rocking (SCR) system, which could be an important addition to the existing rocking pier family. The proposed system aims to mitigate the damage to the main body of the pier and to eliminate residual deformation after earthquakes. These research results have guidance for future research on the application of SMA and SMA-reinforced composites in civil engineering.

In this paper, the influence of the position and content of SMA on the flexural properties of SMAHC laminates was studied by numerical simulation and experimental methods. The effectiveness of the model was verified by comparing simulation results with experimental data, and the influence mechanism of SMA on the flexural properties of SMAHC laminates was further revealed.

## 2. Experimental and Numerical Simulation

### 2.1. Materials

**Figure 1:**
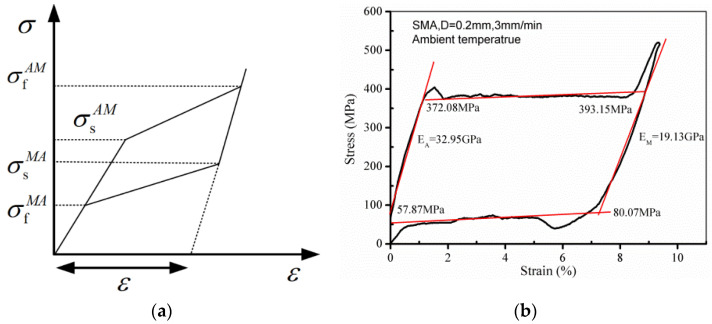
The stress–strain curve of the SMA wire. (**a**) Ideal. (**b**) Experimental.

The matrix is a polymer mixed with thermosetting epoxy resin, accelerator and curing agent. The three materials are ethylene oxide resin 411, methyl ethyl ketone peroxide, and cobalt naphthenate, and the mixing ratio is 1:2:0.5%. A unidirectional glass fiber cloth with a thickness of 0.2 mm and a surface density of 200 g/m^2^ is used as a reinforcing material, bought from Tongxiang Mengtai reinforced composite material Co., Ltd. Superelastic Nitinol with a nickel content of 55.9% has a diameter of 0.2 mm, purchased from Jiangyin [34,35]. The theoretical stress–strain curve of the SMA wire is shown in Figure 1.

**Figure 2:**
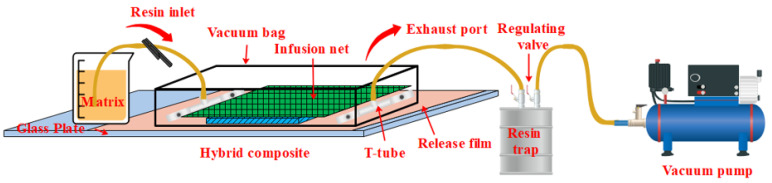
Illustration of VARI.

**Figure 3:**
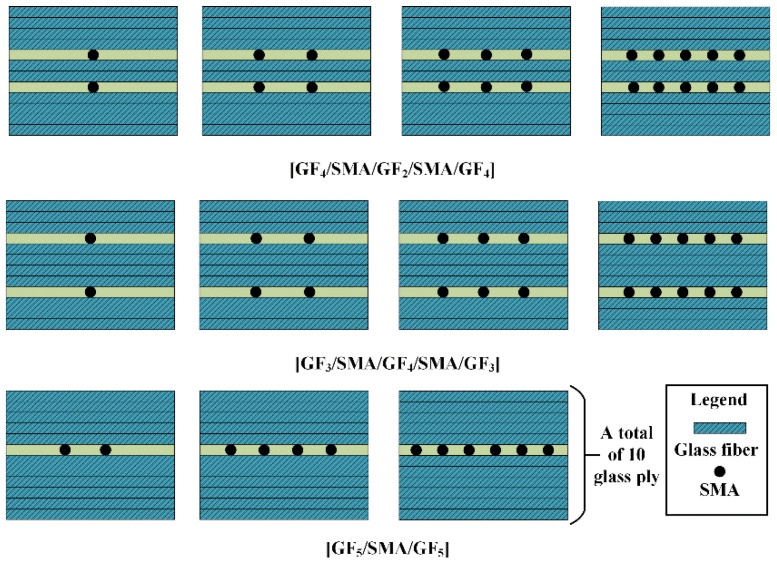
The structural arrangement of SMAHC laminates.

There are three distinct phases in Figure 1a: The purely elastic behavior of the material shows when the stress is less than $\sigma_{s}^{AM}$. The stress is less than $\sigma_{s}^{AM}$, and the material exhibits pure elastic behavior. At critical stress, the forward transition is caused by a stress-induced martensitic transformation (austenite-martensitic). Large transformation strains occur during martensite formation (upper platform). When the stress increases to $\sigma_{f}^{AM}$, SMA is in the martensitic phase, indicating that the forward transformation is completed. Martensite exhibits elastic behavior when the stress exceeds $\sigma_{f}^{AM}$. During unloading, the reverse phase transition from martensite to austenite begins at $\sigma_{s}^{MA}$ and completes at $\sigma_{f}^{MA}$. Due to the difference between $\sigma_{f}^{AM}$ and $\sigma_{s}^{AM}$, and the difference between $\sigma_{s}^{MA}$ and $\sigma_{f}^{MA}$, a hysteresis curve is found in the load/unload stress–strain curve [36,37,38,39,40]. Figure 1b shows the experimental stress–strain curve of the SMA wire. Table 1 shows the material parameters of the SMA wire [41]. Vacuum-assisted resin injection (VARI) was used to prepare composite laminates, as shown in Figure 2, with vacuum curing at room temperature for 9–12 h. The laminate is composed of 10 layers of glass fiber cloth. The structural arrangement of SMAHC laminates is shown in Figure 3. The size of the specimen is 80 mm × 25 mm × 2.4 mm. The number of flexural specimens is given in Table 2. In Table 2, different types of specimens are numbered according to the ply form of bending specimens. B-1, B-2, B-3 and B-4 represent the type of specimen, and B-2-2/4/6/10 represents the embedding of 2/4/6/10 SMA in B-2 specimens, respectively.

### 2.2. Three-Point-Bending Tests

**Figure 4:**
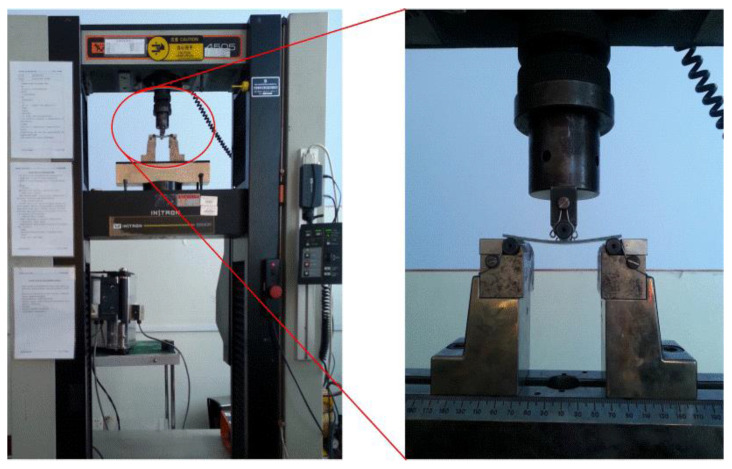
Loading device for bending tests.

The three-point-bending test specimens of SMAHC were prepared according to the ASTM-D790 standard. The INSTRON 4505 universal testing system was used to conduct three-point-bending experiments on composite laminates with different SMA embedment contents and embedment positions at room temperature [42]. Five specimens of each laminated composite plate were prepared, and the loading speed was 3 mm/min. Loading stopped when the specimen was damaged. According to the ASTM-D790 standard, the test is terminated when the maximum strain in the outer surface of the test specimen has reached 0.05 mm/mm or at break if a break occurs before reaching the maximum strain. If no break has occurred in the specimen by the time the maximum strain in the outer surface of the test specimen has reached 0.05 mm/mm, the test is discontinued. The loading of the bending test is shown in Figure 4.

The flexural stress corresponding to any point on the load–deflection curve can be calculated using the following Equation (1):

(1) $$\sigma_{f}=\frac{3PL}{2bd^{2}}$$

where $\sigma_{f}$ is the stress in the outer fibers at the midpoint, *P* is the load at a given point on the load–deflection curve, *L* is the length of the support span, *b* is the width of the tested specimen, and *d* is the depth of the tested specimen.

Nominal fractional change in the length of an element of the outer surface of the test specimen at midspan, where the maximum strain occurs. It may be calculated for any deflection using Equation (2):

(2) $$\varepsilon_{f}=6Dd/L^{2}$$

where $\varepsilon_{f}$ is strain in the outer surface, *D* is maximum deflection of the center of the beam, *L* is support span, and *d* is depth.

### 2.3. Finite Element Modeling

**Figure 5:**
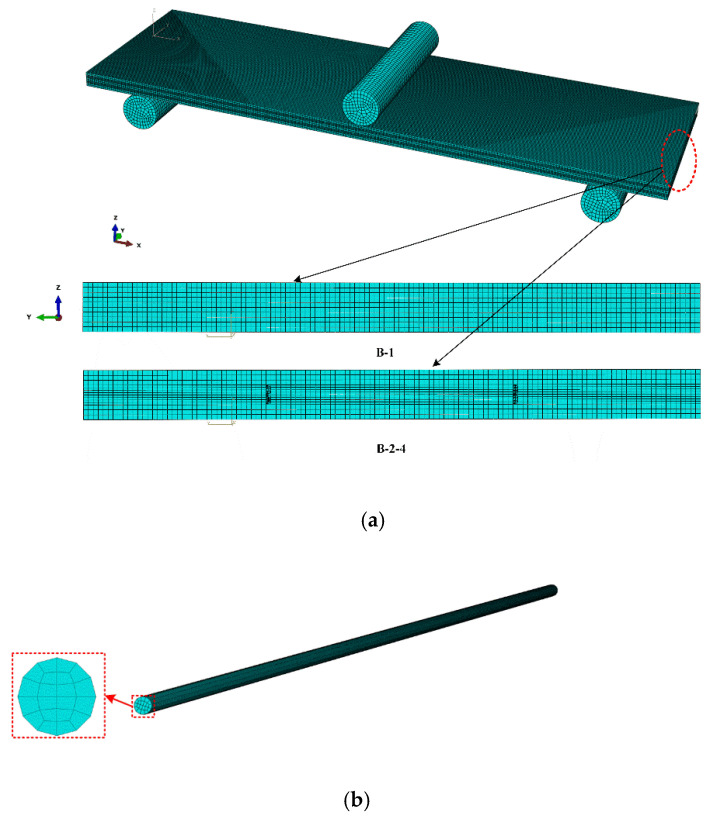
Finite element model of composite laminates. (**a**) The meshing of composite laminates, and (**b**) the meshing of SMA.

Finite element software ABAQUS was used to establish a model to analyze the bending performance of SMAHC. The three-dimensional continuum element C3D8R was used to mesh glass fiber-reinforced composites. The meshing of composite laminates is given in Figure 5a. The meshing of composite laminates is given in Figure 5a. The cylindrical rigid body in the central part of the laminate represents the indenter to apply the load, and the cylinders on both sides of the lower part of the laminate represent the base of the three-point-bending test. These three cylindrical rigid bodies are defined as rigid bodies. The contact between the rigid body and the upper and lower surfaces of the laminate is defined as surface-to-surface universal contact (Figure 5a). The mesh of SMA wire adopts 16,000 linear hexahedral elements of type C3D8R, as shown in Figure 5b. A glass fiber layer with a thickness of 0.2 mm for each layer is adopted, and a total of 10 layers of laminates are used. The layering sequence is ${\left[0{}^{\circ}\right]}_{10}$. The size of the specimen is 80 mm × 25 mm × 2.4 mm. In this paper, the laminated plate specimens are SMAHC laminated plates with one or two layers of SMA wire and ten layers of glass fiber cloth. Mechanical property parameters of GFRP required for finite element analysis are illustrated in Table 3.

To predict the damage of composite materials, such as matrix tensile failure, matrix compression failure, fiber tensile failure and fiber compression failure, a composite material damage model based on Hashin [43,44] was adopted, as follows:

Fiber tension $\left(\sigma_{11}\geq 0\right)$

(3) $${\left(\frac{\sigma_{11}}{X_{T}}\right)}^{2}+{\left(\frac{\sigma_{12}}{S_{12}}\right)}^{2}+{\left(\frac{\sigma_{13}}{S_{13}}\right)}^{2}\geq 1$$

Fiber compression $\left(\sigma_{11}<0\right)$

(4) $$\frac{\sigma_{11}}{X_{C}}\geq 1$$

Matrix tension and compression

(5) $${\left(\frac{\sigma_{11}}{2X_{T}}\right)}^{2}+{\left(\frac{\sigma_{12}}{S_{12}}\right)}^{2}+\left(\frac{\sigma_{22}^{2}}{Y_{T}Y_{C}}\right)+\frac{\sigma_{22}}{Y_{T}}+\frac{\sigma_{22}}{Y_{C}}\geq 1$$

where the tensile strength and compressive strength in the longitudinal and transverse directions are $X_{T}$, $X_{C}$, $Y_{T}$ and $Y_{C}$, respectively. The fiber shear strength measured in the 1–3 and 1–2 directions are *S*_13_ and *S*_12_, respectively.

**Figure 6:**
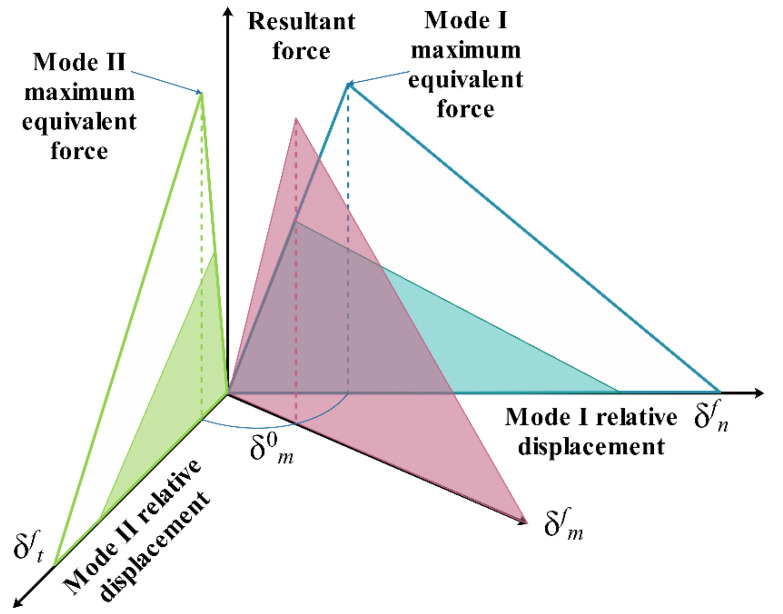
A bilinear mixed-mode traction–separation law.

The surface-based cohesive contact model in ABAQUS was used to simulate the composite effect of laminates [45,46,47]. The interface behavior is governed by a mix-mode bilinear traction–separation law, which assumes an initially linear elastic response followed by the linear evolution of damage, as displayed in Figure 6.

## 3. Results and Discussion

### 3.1. Experimental Results

**Figure 7:**
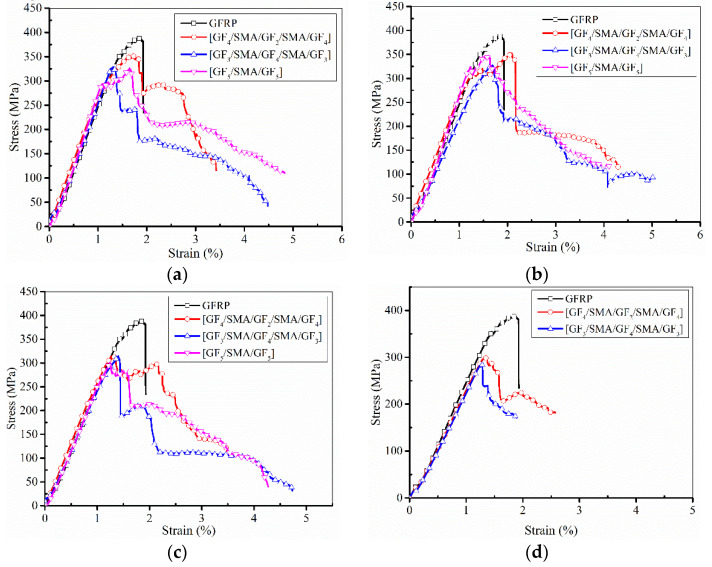
Comparison of the flexural stress-strain curves of different types of SMAHC laminates and GFRP. (**a**) Two wires, (**b**) four wires, (**c**) six wires, (**d**) ten wires.

Figure 7 shows the flexural stress–strain curves of different types of SMAHC and GFRP specimens. It can be seen from the figure that the curve shows a linear response near the origin, and then a nonlinear response until it breaks. The transition from linear response to nonlinear response is often caused by matrix cracking [15]. In Figure 7, the flexural strength of all SMAHC laminated plate specimens is lower than that of GFRP. This is due to the material difference between the matrix and SMA, which will form a weak interface between SMA and the matrix, resulting in stress concentration on the surface of the SMA wire. All specimens of SMAHC laminates show good ductility, which is also due to the presence of SMA, so that the specimens do not suddenly lose the bearing capacity as with GFRP laminates. It can be observed that SMAHC laminates (B-2-4) have the highest bending performance. The flexural performance of SMAHC laminates is given in Table 4 [27]. The flexural strength of SMAHC laminates (B-2-2/4/6) is 368.62 MPa, 369.17 MPa and 318.73 MPa, respectively. Compared with the GFRP laminate, the flexural strength is decreased by 5.84%, 5.76% and 18.63%, respectively. However, the flexural modulus increased from 22.73 GPa to 23.91 GPa, 23.73 GPa and 23.74 GPa, respectively. In addition, the flexural performance of B-2-6 and B-2-10 laminates decreases more obviously than that of GFRP laminates.

**Figure 8:**
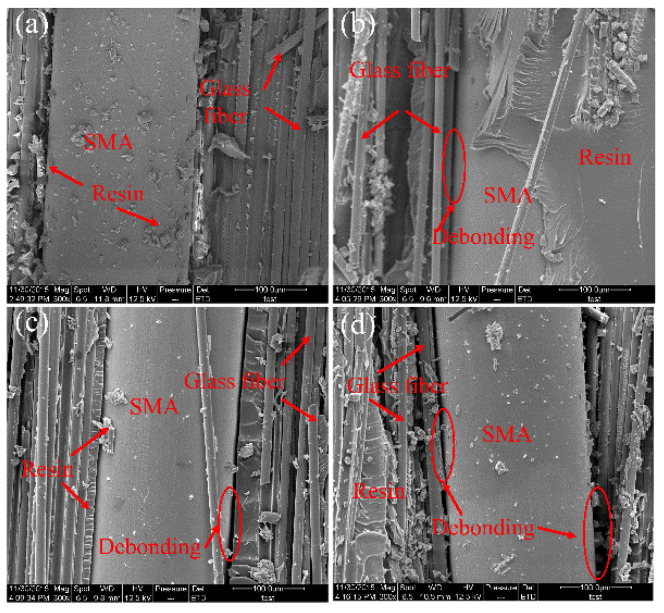
SEM micrograph of the fracture surface of (B-2) specimens. (**a**) B-2-2, (**b**) B-2-4, (**c**) B-2-6, (**d**) B-2-10.

Figure 8 shows SEM micrographs of fracture surfaces of different B-2 specimens. Figure 8a–d corresponds to SEM images at the interfaces of laminated plate specimens (B-2-2/4/6/10) respectively. In Figure 8a, a small amount of resin remains on the surface of the SMA, and the adhesion between the SMA and the matrix is good. As shown in Figure 7b, the resin is wrapped around the SMA well; therefore, the SMA and the matrix are not completely debonded at the fracture surface (B-2-4). In Figure 8c, there is no excessive adhesion of the resin matrix on the surface of the SMA wire, and there is obvious debonding on the tensile side and slight debonding on the compression side. In Figure 8d, the interface between the SMA and the matrix on the compression side and the tensile side is significantly debonded.

**Figure 9:**
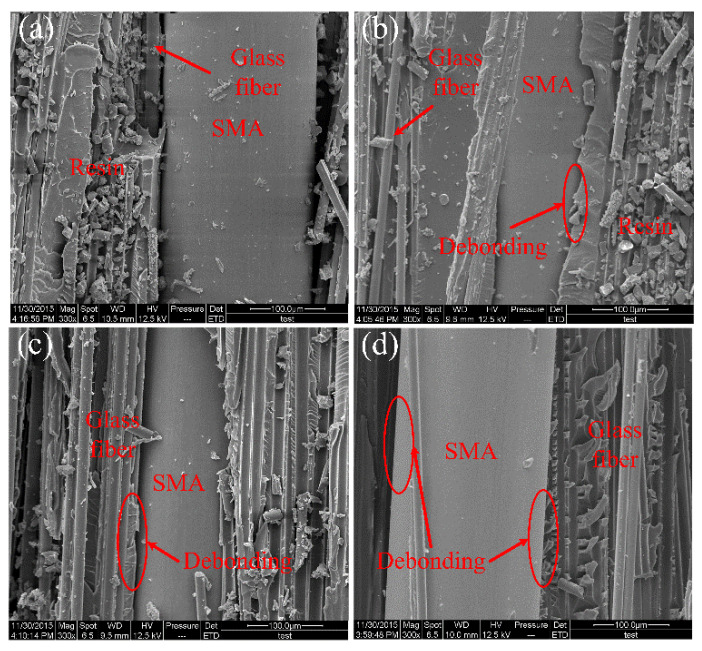
SEM micrograph of the fracture surface of (B-3) specimens. (**a**) B-3-2, (**b**) B-3-4, (**c**) B-3-6, (**d**) B-3-10.

Figure 9 shows SEM micrographs of fracture surfaces of different SMAHC specimens (B-3). The microstructure of the interface between the specimen matrix and SMA wire can be observed in Figure 9. Figure 9a–d corresponds to SMAHC specimens (B-3-2/4/6/10). In Figure 9a, only a small amount of resin remains on the surface of the SMA, and significant debonding is observed between the matrix and the SMA. SEM micrograph of the SMAHC specimen (B-3-4) is shown in Figure 9b. SMA is well-wrapped by the resin matrix and only slightly debonded, indicating that the interface at the fracture surface has no obvious negative effect on the degradation of the specimen properties during loading. In Figure 9c, there is obvious debonding between SMA and matrix, and the glass fiber is broken and part of the fiber is pulled out. As shown in Figure 9d, complete debonding occurs between SMA and matrix, indicating that the specimen has completely failed at this time.

**Figure 10:**
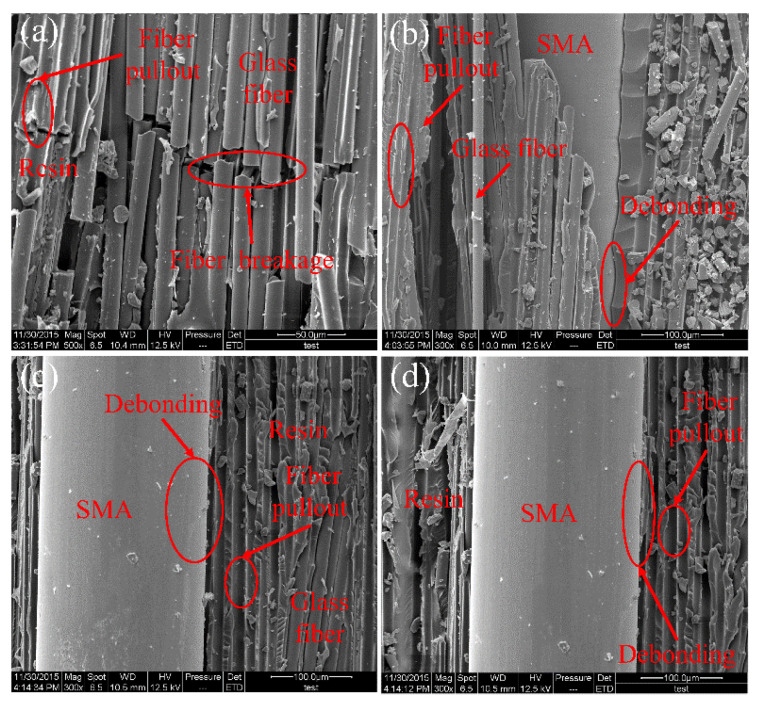
SEM micrograph of the fracture surface of (B-4) specimens. (**a**) B-1, (**b**) B-4-2, (**c**) B-4-4, (**d**) B-4-6.

Figure 10 shows the SEM micrograph of the fracture surface of the GFRP and the SMAHC specimens (B-4). In Figure 10a, the glass fiber is pulled off, and traces of the glass fiber being pulled out can be observed. SEM image of the SMAHC specimen (B-4-2) is shown in Figure 10b. Only slight debonding occurs between SMA and matrix, and SMA is almost completely wrapped by matrix, indicating that the weak interface between matrix and SMA does not play a leading role in the failure process of the specimen. In Figure 10c,d, 4 to 6 SMA wires are embedded in the same layer, and the matrix and SMA are completely debonded and fibers are pulled out. There is no SMA wire to provide tension in the tensile zone of the specimen, and in the loading process, because of too much SMA embedded in the same layer, cracks will occur at the interface and rapidly expand, resulting in the deterioration of the overall performance of the specimen. SEM observation shows that the main failure modes of SMAHC laminates are delamination and debonding, and the mechanical properties of SMAHC laminates are greatly affected by these two failure modes.

### 3.2. Numerical Results

**Figure 11:**
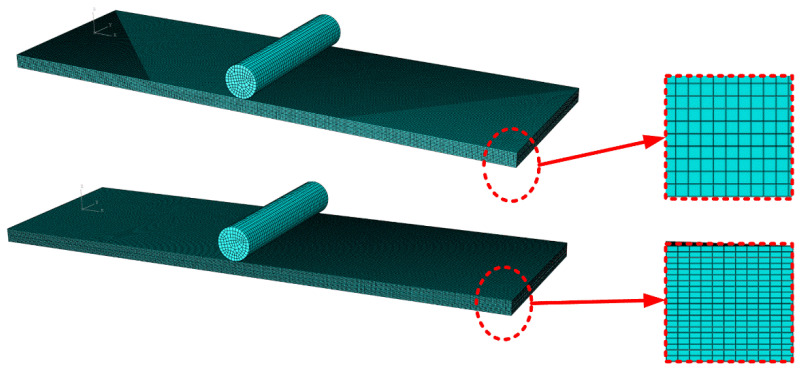
Finite element model of GFRP laminates.

**Figure 12:**
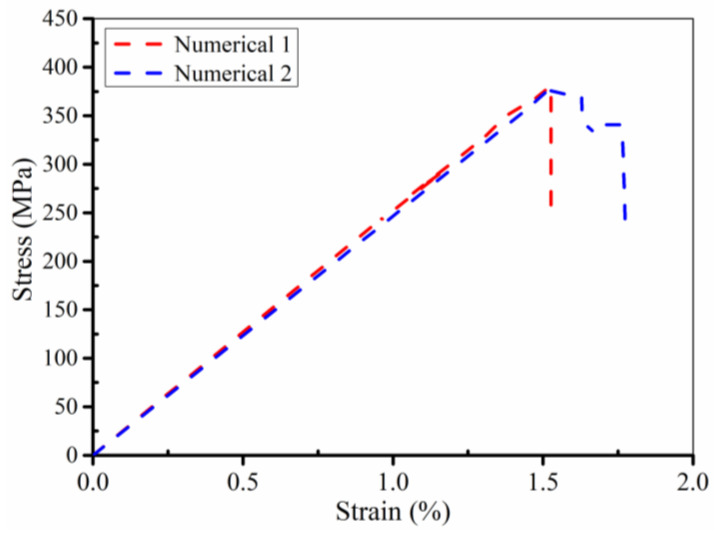
Comparison of GFRP laminates between original and new model.

The convergence of the numerical simulation of the model is tested. The number of elements in the original model is 50,000, and the number of elements in the new model is 100,000. The new model only adds elements in thickness. Figure 11 presents two models. A comparison of GFRP laminates between the original and new model is shown in Figure 12. The new flexuous strength and flexuous modulus of GFRP laminates (B-1) are 375.65 MPa and 24.73 GPa, respectively. The errors of simulation results and experimental results are 1.59% and 1.47%, respectively, which meet the requirements of accuracy.

**Figure 13:**
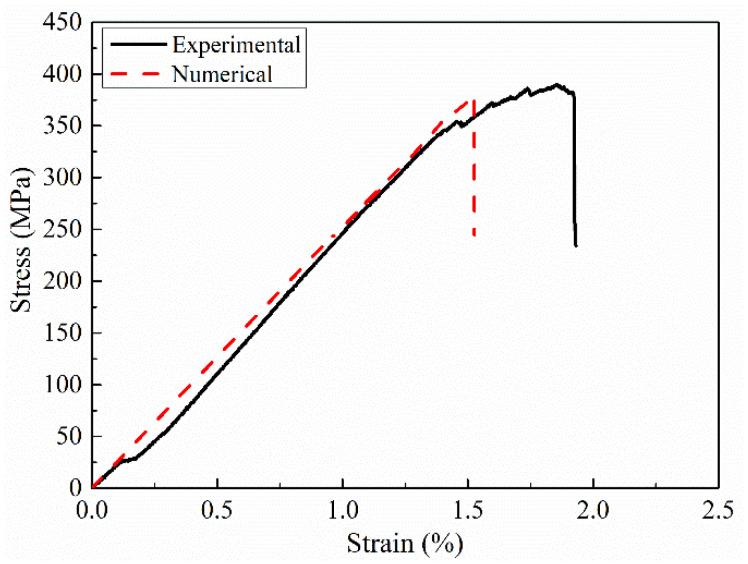
Comparison of GFRP laminates between simulation and experimental.

**Figure 14:**
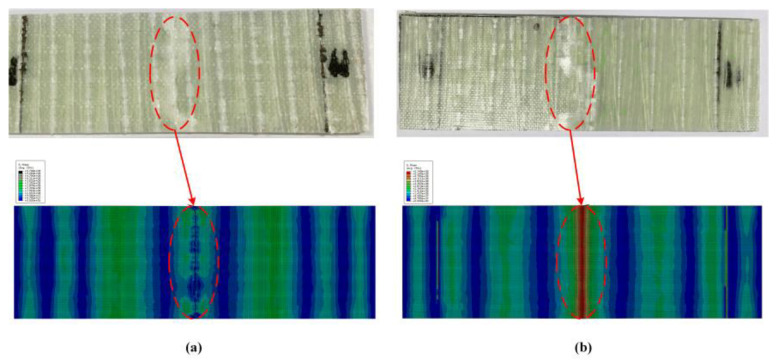
The failure mode of GFRP laminate. (**a**) Compression side, (**b**) Tension side.

The comparison between the stress–strain curve obtained from the bending test of GFRP laminates and the simulation results is shown in Figure 13. It is found that the macroscopic stress–strain curves obtained from experimental tests are basically consistent with those obtained from simulation. The simulated flexural strength and flexural modulus of GFRP laminates (B-1) are 381.71 MPa and 25.1 GPa, respectively. The errors of simulation results and experimental results are 2.56% and 10.43%, respectively, which meet the requirements of accuracy. The experimental and simulation results of GFRP laminates in failure are given in Figure 14. In Figure 14a, the resin matrix on the compression side of the laminate is crushed and a white indentation appears, which is consistent with the simulated failure mode. However, in the tensile side of the laminated plate (Figure 14b), no obvious damage to the matrix occurred in the experimental results and simulation results.

**Figure 15:**
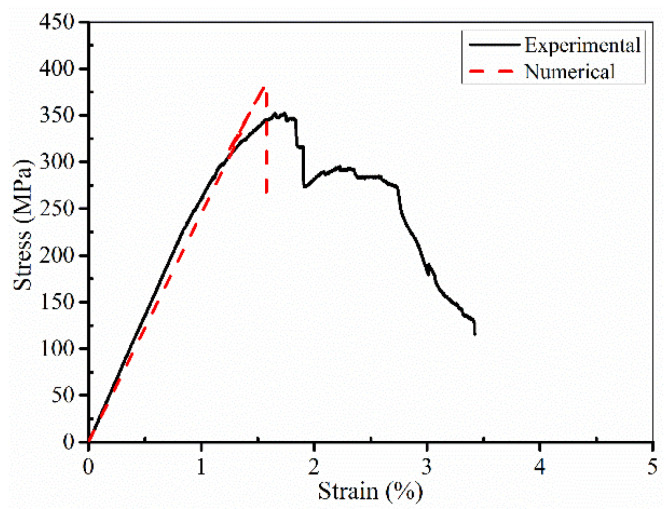
Comparison of SMAHC laminates between simulation and experimental.

**Figure 16:**
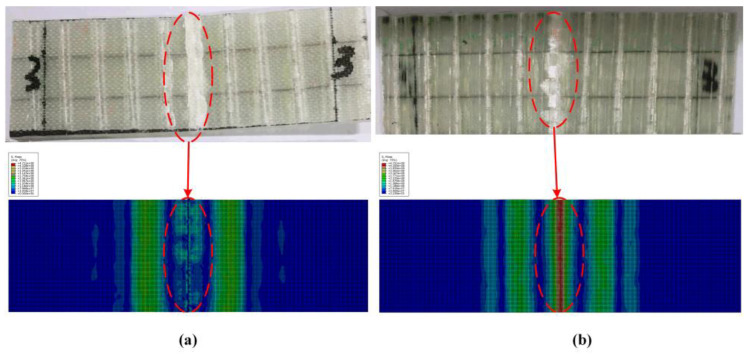
The failure mode of SMAHC laminate. (**a**) Compression side, (**b**) Tension side.

**Figure 17:**
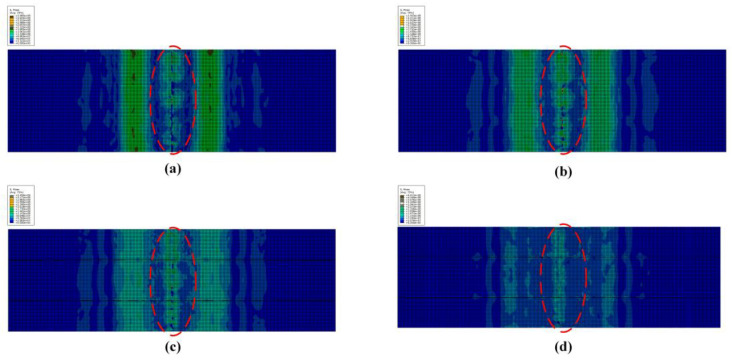
Damage contours of layers 2–5 above the neutral layer of SMAHC laminates. (**a**) Second layer, (**b**) Third Layer, (**c**) Fourth layer, (**d**) Fifth layer.

Figure 15 shows the comparison curve between simulation results and experimental results of SMAHC laminates (B-2-4). The stress–strain curve obtained by experiment is in good agreement with the simulation results. Because of the more serious debonding between SMA and matrix during simulation, the specimen directly loses the bearing capacity without the phenomenon of sustained bearing in the experiment. The flexural strength and flexural modulus of SMAHC laminates (B-2-4) are 385.335 MPa and 24.47 GPa, respectively. The errors of simulation results and experimental results are 4.38% and 2.34%, respectively, which meet the requirements of accuracy. In Figure 16a, because the resin matrix is crushed and the experimental laminate specimen is obviously damaged, the simulation result is consistent with the experimental phenomenon, and obvious damage occurred in the compression region. In the tension side of the laminated plate (Figure 16b), no obvious damage occurred in the matrix in the simulation results, but slight damage occurred in the experimental results. Figure 17 shows the damage profile of two to five layers above the neutral layer of SMAHC laminates. It can be clearly observed in Figure 16 that obvious damage also occurred in the second layer, while slight damage occurred in the third layer, and no obvious damage occurred in the fourth and fifth layers. This is because the upper side of the neutral layer is the compression side, the matrix is damaged under pressure, and the existence of the weak interface between SMA and the matrix will occur debonding damage, then lead to the lamination of the laminated plate, the laminated plate, as a whole, loses bearing capacity, so the fourth layer and the fifth layer of the matrix damage is not obvious. It is important to note that the model, here, does not take into account the degradation of SMA functionality, and it does have some limitations for a wider range of applications.

## 4. Conclusions

In this paper, the flexural behavior of SMAHC laminates was studied experimentally and numerically. The experimental results and simulation results were compared to verify the validity of the model. The main conclusions obtained were made as follows:

1. The optimal number of SMA wires for SMAHC laminates (B-2) is four. Compared with GFRP laminates, the flexural modulus of B-2-4 laminates increases by 5.19%, while the strength decreases by 5.76% on average. The flexural properties of B-2-6 and B-2-10 laminates decrease more obviously, and the weak interface between fiber and matrix is the main reason for the degradation of the flexural properties.
2. The microstructure shows that the material difference between SMA and matrix forms a weak interface, which produces microcracks and expands rapidly during the loading process. The interface debonding failure results in matrix delamination and the final specimen failure. The weak interface between the matrix and SMA increases with the increase of the number of SMA wires, which is convenient for the formation of matrix delamination damage. The main failure modes of SMAHC composites are delamination and debonding, and this failure mode has an obvious influence on the mechanical properties of the composites.
3. The finite element models of GFRP and SMAHC (B-2-4) laminates were established and compared with the experimental results. The numerical prediction results were in good agreement with the experimental results. The errors between the simulation results of GFRP laminates and the experimental results of flexural performance were 2.56% and 10.43%, respectively. The errors of experimental results and simulation results of the flexural performance of SMAHC laminates (B-2-4) are 4.38% and 2.34% respectively, which meet the accuracy requirements and verify the validity of the model.

## Figures and Tables

**Table 1:** Material parameters of SMA wire [41].

Material Parameter	Martensite	Austenite
Phase transformation stress (MPa)	$\sigma_{f}^{AM}$ = 393.15, $\sigma_{s}^{AM}$ = 372.08	$\sigma_{f}^{MA}$ = 57.89, $\sigma_{s}^{MA}$ = 80.07
Phase transformation strain (%)	*ε* = 0.94, *ε* = 9.41	*ε* = 7.09, *ε* = 0.35
Elasticity modulus (GPa)	$E_{M}$ = 19.13	$E_{A}$ = 32.95
Tensile strength (MPa)	1115.26	
Failure strain (%)	12	

**Table 2:** Tensile and bending test specimens.

Specimen	Number
[GF_10_]	B-1
[GF_4_/SMA/GF_2_/SMA/GF_4_]	B-2-2/4/6/10
[GF_3_/SMA/GF_4_/SMA/GF_3_]	B-3-2/4/6/10
[GF_5_/SMA/GF_5_]	B-4-2/4/6

**Table 3:** Material properties of GFRP laminates.

$E_{11}$ (GPa)	$E_{22}=E_{33}$ (GPa)	$\nu_{12}=\nu_{13}$	$\nu_{23}$	$G_{12}=G_{13}$ (GPa)	$G_{23}$ (GPa)
24.43	7.11	0.3	0.34	6.34	1.41
$X_{T}$ (MPa)	$X_{C}$ (MPa)	$Y_{T}=Z_{T}$ (MPa)	$Y_{C}=Z_{C}$ (MPa)	$S_{12}=S_{13}$ (MPa)	$S_{23}$ (MPa)
666.44	320.44	70.8	188.9	51.7	51.7

**Table 4:** Flexural properties of different types of SMAHC laminates.

Specimen	Number	0	2	%	4	%	6	%	10	%
B-2	Modulus (GPa)	22.73	23.81		23.91 ↑		22.42		22.11	
		± 0.37	± 0.4	4.75	± 0.35	5.19	± 0.41	−1.32	± 0.31	−2.73
	Strength (MPa)	391.74	368.86		369.17		318.73		309.23	
		± 6.94	± 6.5	−5.84	± 5.2	−5.76	± 6.4	−18.63	± 6.9	−21.06
B-3	Modulus (GPa)	22.73	23.61		23.73 ↑		22.38		21.66	
		± 0.37	± 0.39	3.87	± 0.35	4.40	± 0.42	−1.54	± 0.32	−4.71
	Strength (MPa)	391.74	334.67		342.57		316.68		289.40	
		± 6.94	± 4.14	−14.57	± 4.56	−12.55	± 6.51	−19.16	± 5.82	−26.12
B-4	Modulus (GPa)	22.73	22.74		23.74↑		22.21		-	
		± 0.37	± 0.3	0.04	± 0.32	4.4	± 0.31	−2.89		
	Strength (MPa)	391.74	349.69		313.85		304.95		-	
		± 6.94	± 5.35	−10.73	± 4.2	−19.88	± 6.1	−22.16		

## Data Availability

Not applicable.
